# Protein oxidative damage in the hippocampus in a mouse model of acute hyperammonemia

**DOI:** 10.1186/2047-783X-19-S1-S29

**Published:** 2014-06-19

**Authors:** Jasmin Klose, Boris Goerg, Carsten Berndt, Dieter Häussinger, Orhan Aktas, Tim Prozorovski

**Affiliations:** 1 Department of Neurology, Heinrich Heine University, 40225 Düsseldorf, Germany; 2 Clinic of Gastroenterology, Hepatology and Infectious Diseases, Heinrich Heine University, 40225 Düsseldorf, Germany

## 

Hepatic encephalopathy (HE) is a common neuropsychiatric complication of both chronic and acute liver failure that is characterized by psychomotor, intellectual and cognitive impairment [1]. The mechanism of neuronal dysfunction in HE has not yet been conclusively investigated and possibly implies numerous pathological processes. It is widely accepted that among the factors contributing to HE, hyperammonemia, due to ammonia dysmetabolism and inflammation as a response to infection, play a major role in the pathogenesis. A growing body of evidence, e.g. RNA oxidation and protein nitrosylation, further indicate that oxidative stress occurs in neural cells as a result of ammonia intoxication and may lead to subsequent tissue damage [2,3].

Particular interest of this study was to elucidate the effect of an acute ammonia load on protein homeostasis in the hippocampus, the brain area of learning, memory formation and consolidation. To this end we analysed the accumulation of oxidized proteins, protein ubiquitination, proteasome activity and expression of immunoproteasome subunits after a single intraperitoneal injection of NH_4_Ac (10 mmol/kg body weight) in 6 weeks old C57B/6 mice.

Following ammonia administration the animals fall into coma with a survival rate of 10%. After approximately one hour of coma, the motor ability and reflexes were restored. To analyse whether ammonia intoxication has a delayed effect on CNS tissue, animals were sacrificed 24 hours post injection, perfused with PBS and hippocampi were dissected. Western blot detection of protein carbonylation by OxiBlot technique revealed a significant accumulation of oxidized proteins in the hippocampus of animals with ammonia-precipitated encephalopathy compared to the saline-treated control group. Involvement of oxidative stress in disturbed protein homeostasis was further supported by detection of increased expression of the antioxidant enzymes hemeoxygenase 1 (HO-1) and thioredoxin 1 (Trx1). Similar to the animal model used in this study, up-regulation of HO-1 has been previously described in CNS tissue of patients with HE [4]. Moreover we found that protein oxidation was associated with elevated levels of ubiquitinated proteins and activation of proteasome (chemotrypsin-like) activity, representing the major cellular machinery for degradation of misfolded or damaged proteins. These data indicate that the ubiquitin-proteasome system may play an important role in the cellular response to ammonia-induced protein damage. To analyse whether hyperammonemia has a direct effect on neural cells, in an *in vitro* approach we exposed mixed glial cultures and the BV2 microglial cell line to 5 mM NH_4_Cl. Here, comparable to our findings in animals with ammonia-induced encephalopathy, 24 hours treatment increased the total amount of oxidized and ubiquitinated proteins, proteasome activity and expression of antioxidant enzymes. Notably, ammonia-induced protein damage was associated with a diminished functional role of microglia in terms of myelin debris clearance as it was observed using a myelin phagocytosis assay. Treatment with the antioxidant compound *N-acetylcysteine* rescued the effect of ammonia on impaired myelin engulfment and accumulation of ubiquitinated proteins.

Taken together, our data show that ammonia intoxication induces protein oxidation in hippocampal tissue. This result corresponds to findings of other groups indicating impaired long-term potentiation in the hippocampus of animals with hyperammonemia and impaired cognitive function. We observed that an alteration of the protein homeostasis may persist longer after initial onset of neurological deterioration and thus, may contribute to a delayed effect of ammonia toxicity on neuronal activity. Upregulation of anti-oxidant enzymes in both mouse experimental hyperammonemia and patients with HE indicates that similar oxidative processes may underlie the CNS tissue damage. Induction of proteasome activity observed in this study may have a crucial role in the cellular adaptation in response to disturbed protein homeostasis. Moreover, our data indicate that hyperammonemia alone is able to affect microglia (the brain’s innate immune cells) and may have a negative impact on homeostatic and/or inflammatory function of microglia during the course of hepatic encephalopathy.
